# Whole Genome Resequencing Reveals High Genetic Homogeneity and Localized Candidate Differentiation Signals in *Saxidomus purpuratus* Along the Yellow and Bohai Sea

**DOI:** 10.3390/ijms27219627

**Published:** 2026-10-27

**Authors:** Shuai Xu, Chunnuan Zhao, Haiying Han, Yuping Wu, Liqun Ren, Biao Wu, Chaoyi Xie, Junjie Li, Tao Yu

**Affiliations:** 1 Changdao Enhancement and Experiment Station, Chinese Academy of Fishery Sciences, Yantai 265800, China; shxu_v@163.com (S.X.); 15865600367@163.com (C.Z.); zzz00101@foxmail.com (H.H.); wuyuping12@foxmail.com (Y.W.); zzzrenliqun@163.com (L.R.); xiecy1104@163.com (C.X.); zzzlijunjie@163.com (J.L.); 2 Island and Reef Fishery Research Center, Chinese Academy of Fishery Sciences, Yantai 265800, China; 3 Yellow Sea Fisheries Research Institute, Chinese Academy of Fishery Sciences, Qingdao 266071, China; wubiao@ysfri.ac.cn

**Keywords:** *Saxidomus purpuratus*, whole-genome resequencing, genetic diversity, population structure, localized candidate differentiation

## Abstract

*Saxidomus purpuratus* (*S. purpuratus*) is an economically important bivalve along the northern coast of China, where intensifying fishing pressure threatens its wild stocks. Its genome-wide population genetic variation remains poorly characterized. We resequenced 75 wild individuals from five populations spanning the northern Yellow Sea (Donggang, DDDG; Ganjingzi, DLGJ; Huancui, WHHC) and the Bohai Sea (Changdao, YTCD; Laizhou, YTLZ). The sampled populations exhibited relatively low genetic diversity (He = 0.1301–0.1324), weak among-population differentiation (Fst = 0.0035–0.0040), and heterozygosity deficiency (Fis = 0.0480–0.0831). PCA, NJ, and ADMIXTURE detected no geographic structure. PSMC revealed concordant demographic histories, with a sustained decline following a Middle Pleistocene expansion, consistent with historical allele exchange. Selection scans (DDDG as the target population; WHHC and YTCD as references) identified 1268 candidate genes, of which 197 were shared across the two comparisons. After masking a tandem histone gene cluster, the remaining 155 non-histone genes fell into five functional categories: receptor and signal transduction, membrane transport and ion channels, innate immunity and defense, extracellular matrix and adhesion, and detoxification and metabolism. These signals recurred in both comparisons, identifying DDDG as a candidate population for genotype–environment association testing. Together, these results indicate that the five populations constitute a single genetically connected unit, while pinpointing a small set of candidate loci that may underpin local adaptation and should inform stock-enhancement programmes.

## 1. Introduction

Genetic diversity underpins the capacity of populations to adapt to environmental change and to evolve over time [1,2,3,4], and it provides the basis for germplasm evaluation and selective breeding in aquatic species. In marine bivalves, larval dispersal can connect populations despite the limited movement of adults. The spatial pattern of genetic variation is therefore shaped by the interaction of geographic distance, environmental heterogeneity, and harvest pressure [2,5,6]. Robust estimates of genetic diversity, population structure, and demographic history are required to evaluate germplasm resources and to identify potential barriers to gene flow before conservation or stock-enhancement measures are designed [7,8].

*Saxidomus purpuratus* (*S. purpuratus*), a burrowing bivalve, occurs along the coasts of northern China, Japan, and the Korean Peninsula [9,10], where its wild stocks are exposed to increasing harvest pressure that has heightened the need to assess and conserve their genetic resources [11]. Genetic diversity data are essential for evaluating population status and for developing sound conservation and management strategies.

Genetic studies of *S. purpuratus* to date have relied mainly on mitochondrial sequences, molecular markers, and transcriptomic data [9,12,13]. These approaches have yielded information on maternal lineages, marker variation, and gene expression, but their capacity to resolve genome-wide nuclear structure, admixture, and population differentiation is limited. Mitochondrial DNA represents a single maternally inherited locus, and amplified fragment length polymorphism markers capture only a limited fraction of genomic variation [9,13,14]. By contrast, genome-wide SNP data have resolved population-level differentiation associated with local environmental conditions or artificial selection in *Haliotis discus hannai* [1], *Mizuhopecten yessoensis* [7], *Anadara granosa* [15], *Ruditapes philippinarum* [16], and *Crassostrea gigas* [17]. The same datasets capture genetic diversity, demographic history, and population structure within a single analytical framework [3,18,19]. To date, no genome-wide characterization of *S. purpuratus* structure or differentiation has been reported.

*S. purpuratus* is distributed in China mainly from the northern Yellow Sea to the Bohai Sea [10,11]. As a stenohaline, cold-water bivalve, it is sensitive to environmental shifts in these shallow continental-shelf waters. Quaternary glacial–interglacial sea-level oscillations and coastal currents have shaped the distribution and divergence of coastal fauna [6,8,20]. High dispersal potential in marine invertebrates does not preclude local adaptation [5,16,21]. Under strong selection gradients, even taxa with planktonic larval stages can retain signatures of adaptive divergence [6,8,22]. In the Yellow and Bohai Seas, where environmental heterogeneity is pronounced and demographic history is complex, the genetic diversity, population structure, and differentiation of *S. purpuratus* remain to be clarified.

To address this gap, we performed whole-genome resequencing of 75 wild *S. purpuratus* individuals from five geographic populations spanning the northern Yellow Sea (Donggang, DDDG; Ganjingzi, DLGJ; Huancui, WHHC) and the Bohai Sea (Changdao, YTCD; Laizhou, YTLZ). Quantitative traits were recorded for the same individuals. Specifically, we asked whether larval dispersal along regional currents maintains sufficient connectivity for these populations to form a single, weakly differentiated unit; whether the steep temperature–salinity gradient has driven divergent selection at a limited number of loci, producing localized candidate differentiation at the most environmentally distinct site; and whether the five populations share a common demographic history shaped by Quaternary glacial–interglacial sea-level oscillations. We addressed these questions with genome-wide analyses of genetic diversity, population structure, differentiation and demographic history.

## 2. Results

### 2.1. Quantitative Trait Analysis

Quantitative traits differed among populations (Table 1). WHHC exhibited the largest mean shell length, shell height, and wet weight, whereas YTLZ had the greatest mean shell width. DDDG and YTCD were smaller (wet weight ~205 g), 24.5% below that of WHHC. DLGJ ranked second in wet weight and showed the highest shell width/length ratio (0.549) despite its shorter shell, indicating a broader shell and superior body condition. Shell height/length (0.783–0.817) and shell width/length (0.512–0.549) ratios varied little across populations, so morphological differences resided in overall body size rather than in relative proportions. Coefficients of variation were low for the shell dimensions (shell length 3.2–6.2%, shell height 3.7–5.9%) and moderate for wet weight (9.5–14.2%), indicating limited within-population variation in body size. These trait differences alone cannot reveal underlying genetic divergence. To test whether this phenotypic variation has a genetic basis, we examined genome-wide genetic variation (Section 2.2, Section 2.3, Section 2.4, Section 2.5 and Section 2.6).

### 2.2. Sequencing and Genetic Variant Identification

Whole-genome resequencing of 75 *S. purpuratus* individuals from the five populations generated 804.7 GB of clean data at an average depth of 10× and a mean GC content of 35.13% (Table S1). Mean mapping rates to the reference genome ranged from 96.32% to 98.03% across populations. After GATK hard filtering, 112,162,272 variant sites were identified, of which 91,851,824 (81.9%) were biallelic (76,276,475 SNPs and 15,575,349 indels) and 20,310,448 were multi-allelic. Indels were removed, and biallelic SNPs with a minor allele frequency (MAF) > 0.05 and a genotyping rate ≥ 80% (missingness < 20%) were retained. This final set of 20,702,921 SNPs was used for all downstream population-genetic analyses. Functional annotation with ANNOVAR assigned these SNPs to genomic regions (Table S2). Most were intergenic (11,073,890; 53.5%), followed by intronic (6,789,870; 32.8%) and exonic (1,216,750; 5.9%). Among the exonic SNPs, 655,798 (53.9%) were synonymous, 552,121 (45.4%) nonsynonymous, 7651 (0.63%) stop-gain and 1179 (0.10%) stop-loss.

### 2.3. Genetic Diversity and Linkage Disequilibrium

Genome-wide diversity and linkage disequilibrium were first characterized to establish the demographic and linkage context for the subsequent analyses. Genetic diversity indices were similar across the five populations (Table 2). MAF ranged from 0.0699 to 0.0713, with the highest value in DDDG and the lowest in YTLZ. Ho (0.1193–0.1257), He (0.1301–0.1324), and PIC (0.1215–0.1236) followed the same pattern, with higher values in DDDG and lower values in YTLZ. Ho fell below He in every population, and Fis ranged from 0.0480 to 0.0831, indicating that all populations carried some degree of heterozygote deficiency. YTLZ had the highest Fis, approximately 1.7 times the value estimated for DLGJ, and the greatest difference between Ho and He. Over short genetic distances, r^2^ fell from 0.3882–0.3893 to below 0.2 in all five populations before approaching comparable plateau levels (Figure S1). The YTLZ curve declined from 0.3889 to 0.1871; its decay was slightly faster, but the magnitude of the difference was small.

### 2.4. Genetic Structure and Differentiation of S. purpuratus Populations

**Figure 1:**
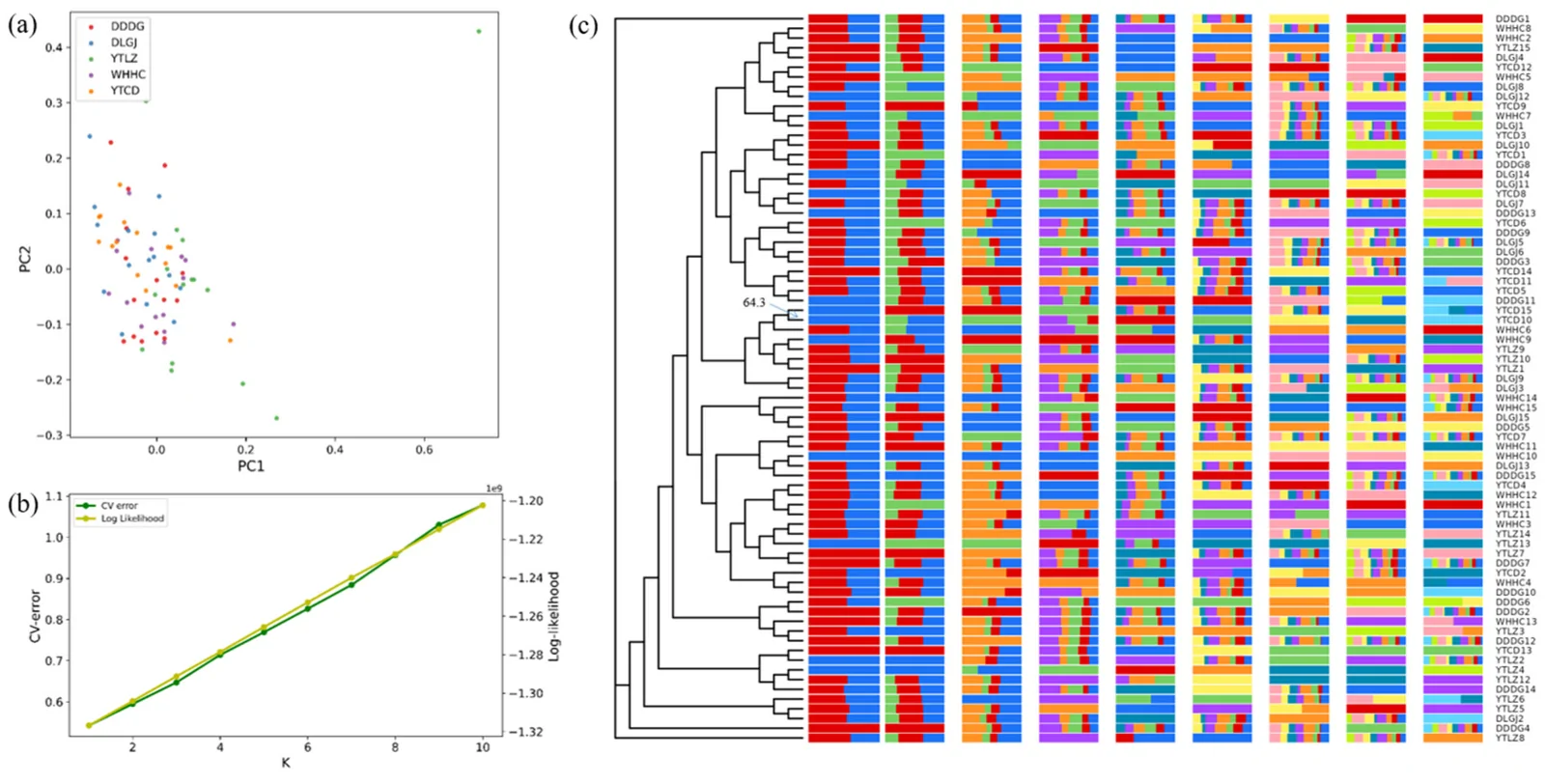
Population relationships and genetic structure among five populations. (**a**) Principal component analysis (PCA). (**b**) Best K-value with Admixture Cross-validation. (**c**) Neighbor-joining tree and population structure analysis. Bootstrap support values (1000 replicates) ≥50% are shown at the nodes.

To determine whether the five populations are genetically connected, we examined population structure (PCA, ADMIXTURE, and NJ) and pairwise Fst. Individuals from all populations overlapped along PC1 and PC2, with no population confined to a discrete region of the ordination space (Figure 1a). YTLZ lay at the greatest distance from the overall centroid, although this separation remained within the range of variation among individuals. The NJ tree interspersed individuals from different populations across short branches (Figure 1c). No sampled population resolved as monophyletic, and the peripheral branch containing YTLZ did not define an independent lineage. Bootstrap support was low across most nodes of the NJ tree (only a single node exceeded 50%), consistent with the weak population structure. ADMIXTURE assigned the lowest cross-validation error (0.54) at K = 1 (Figure 1b). Ancestry components inferred at higher K bore no stable relationship to geographic origin, and the component predominant in YTLZ at K = 2 fell short of constituting a distinct ancestry group.

Pairwise Fst values ranged from 0.0035 to 0.0040 across the ten population comparisons (Table S3). The value for YTCD and DDDG, which are located in different seas, was 0.0037. A similar value (0.0038) was obtained for WHHC and DDDG within the Yellow Sea. Geographic separation between populations in the Yellow Sea and the Bohai Sea was not apparent at the spatial scale examined. The 95% confidence intervals of pairwise Fst, derived from 1000 window-based bootstrap replicates, did not include zero, indicating weak but statistically significant differentiation.

### 2.5. Gene Flow and Population History

**Figure 2:**
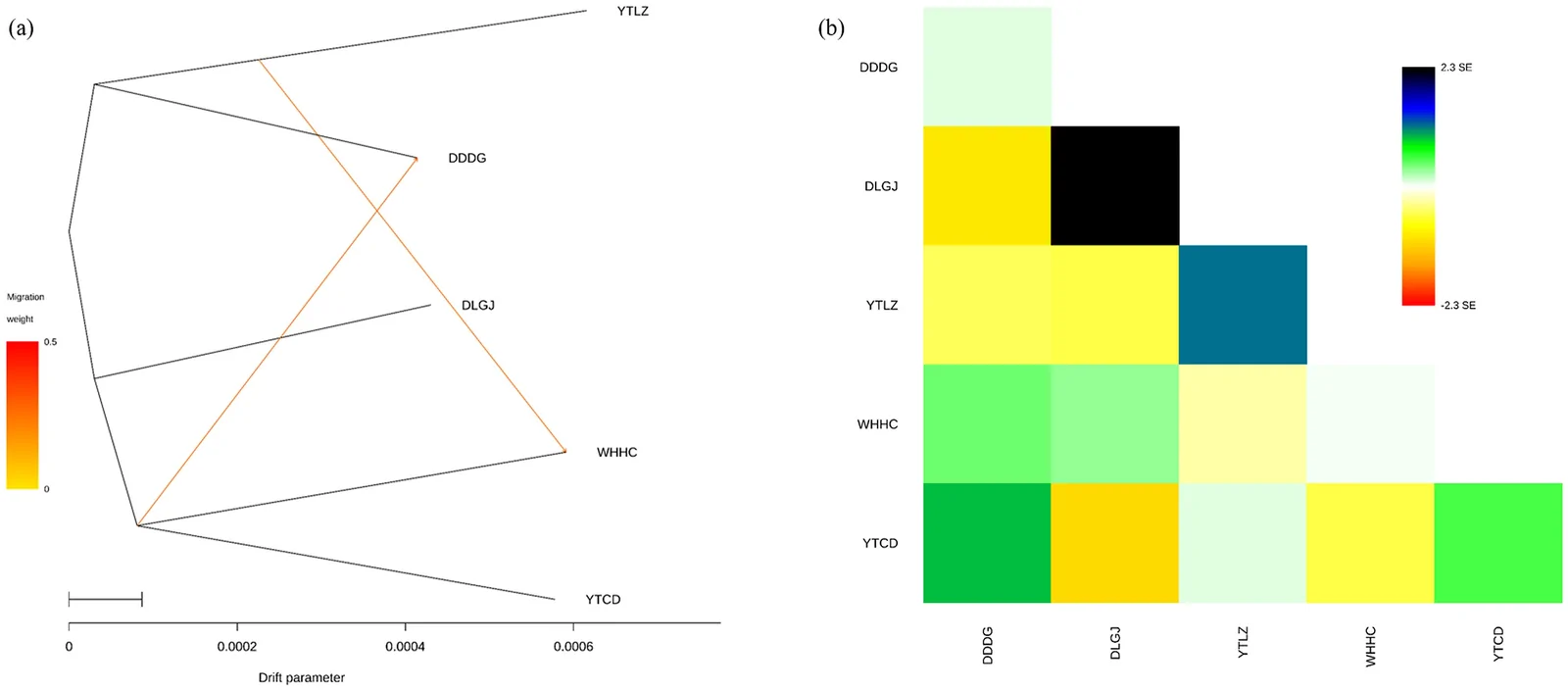
Inference of gene flow among five *S. purpuratus* populations using TreeMix. (**a**) Population diffusion patterns are shown as black lines, directions are indicated by arrows, and colors represent intensities. (**b**) Residual fit of migration events.

Shared demographic history was investigated by reconstructing gene flow and population-size trajectories with TreeMix and PSMC. The TreeMix tree placed DDDG on the earliest branch, followed by YTCD, whereas DLGJ, WHHC, and YTLZ formed a cluster with YTLZ at the distal position (Figure 2a). OptM selected a model with two migration edges, which explained approximately 92% of the sample covariance and produced small residuals between population pairs (Figure S2 and Figure 2b). The branching order has limited interpretive power because genome-wide differentiation was very low. The migration edges identify covariance patterns compatible with historical allele exchange.

**Figure 3:**
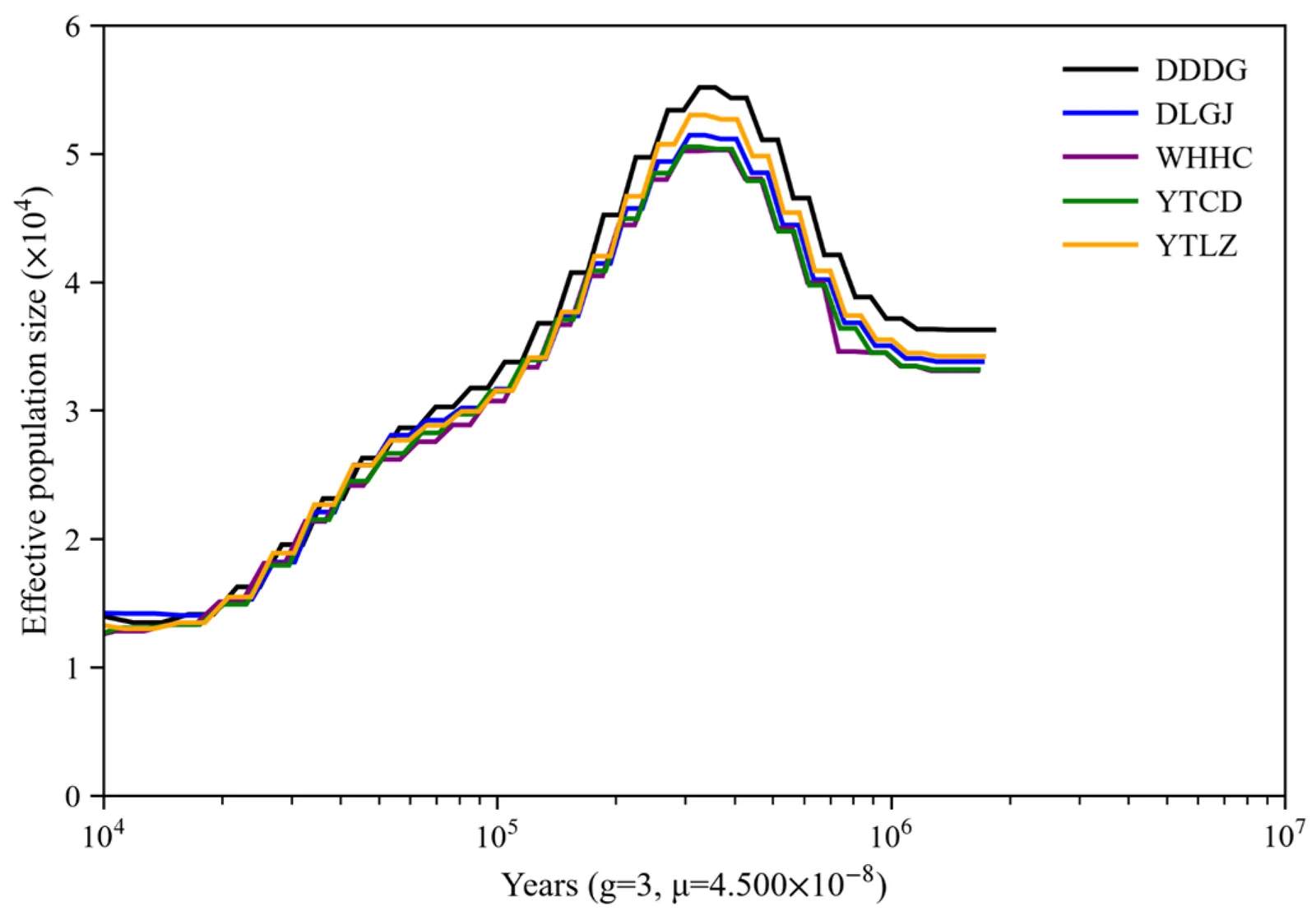
Effective population size of five *S. purpuratus* populations.

Before approximately 1× 10^6^ years ago, the estimated Ne remained between 3.0 × 10^4^ and 3.6 × 10^4^. During the Middle Pleistocene, approximately 3 × 10^5^ to 5 × 10^5^ years ago, Ne increased to 5.0 × 10^4^–5.5 × 10^4^. A prolonged decline followed through the Late Pleistocene, with estimates reaching 1.0 × 10^4^–1.3 × 10^4^ by ~10^4^ years ago (Figure 3). These concordant trajectories are compatible with a shared demographic history. PSMC alone cannot distinguish recent gene flow from shared ancestral population history or establish reproductive isolation. Estimates younger than ~1 × 10^4^ years BP lie below the resolution limit of PSMC (~2 Ne·g) and were not interpreted as contemporary effective population size.

### 2.6. Detection of Candidate Differentiated Regions and Functional Enrichment

**Figure 4:**
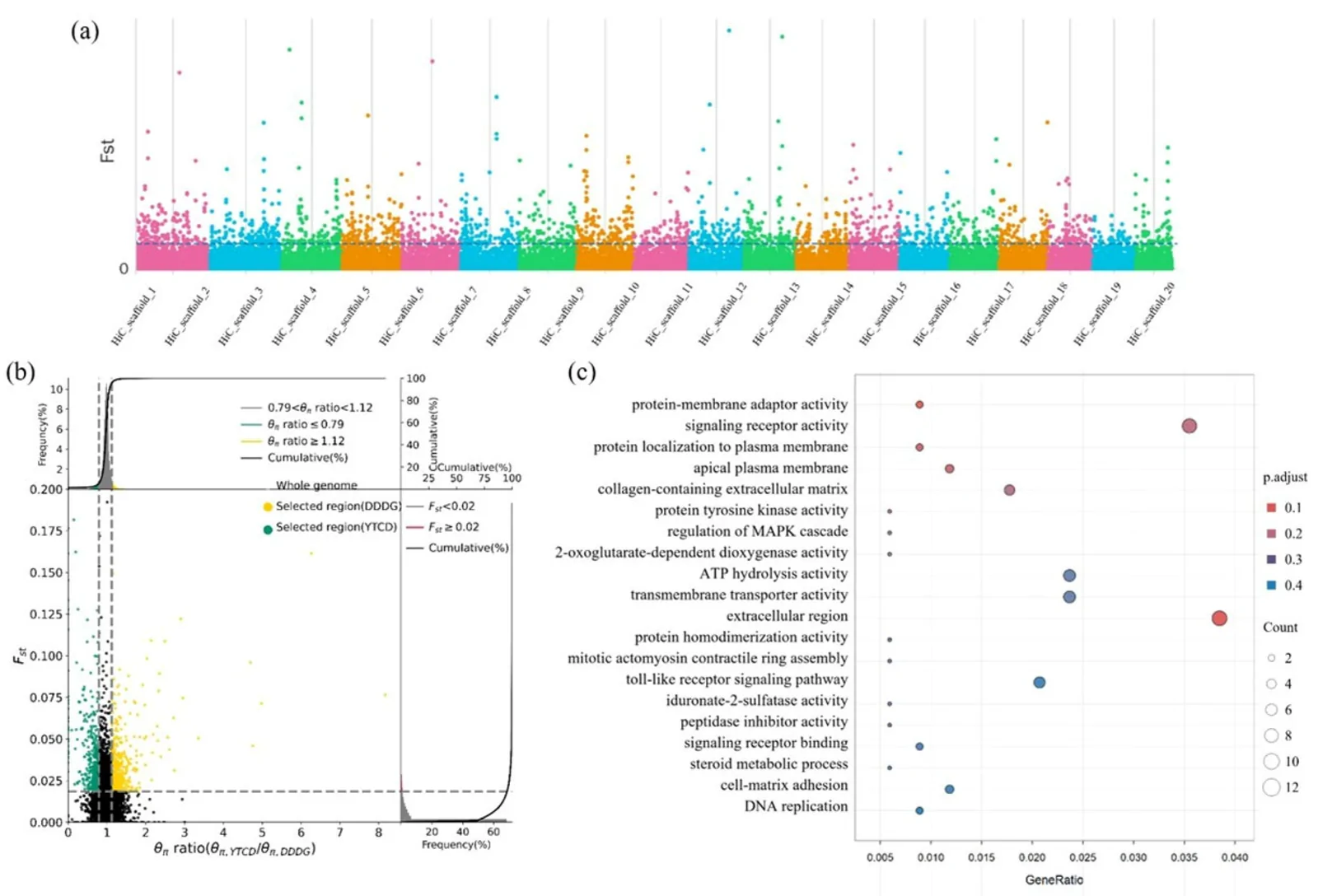
Selective sweep signals and GO enrichment analysis of *S. purpuratus* (YTCD vs. DDDG). (**a**) The values of Fst between YTCD and DDDG were plotted against the position on each HiC_scaffold. (**b**) Distribution of Fst values and θπ ratio calculated between YTCD and DDDG. (**c**) GO term analysis of selected genes in YTCD and DDDG.

**Figure 5:**
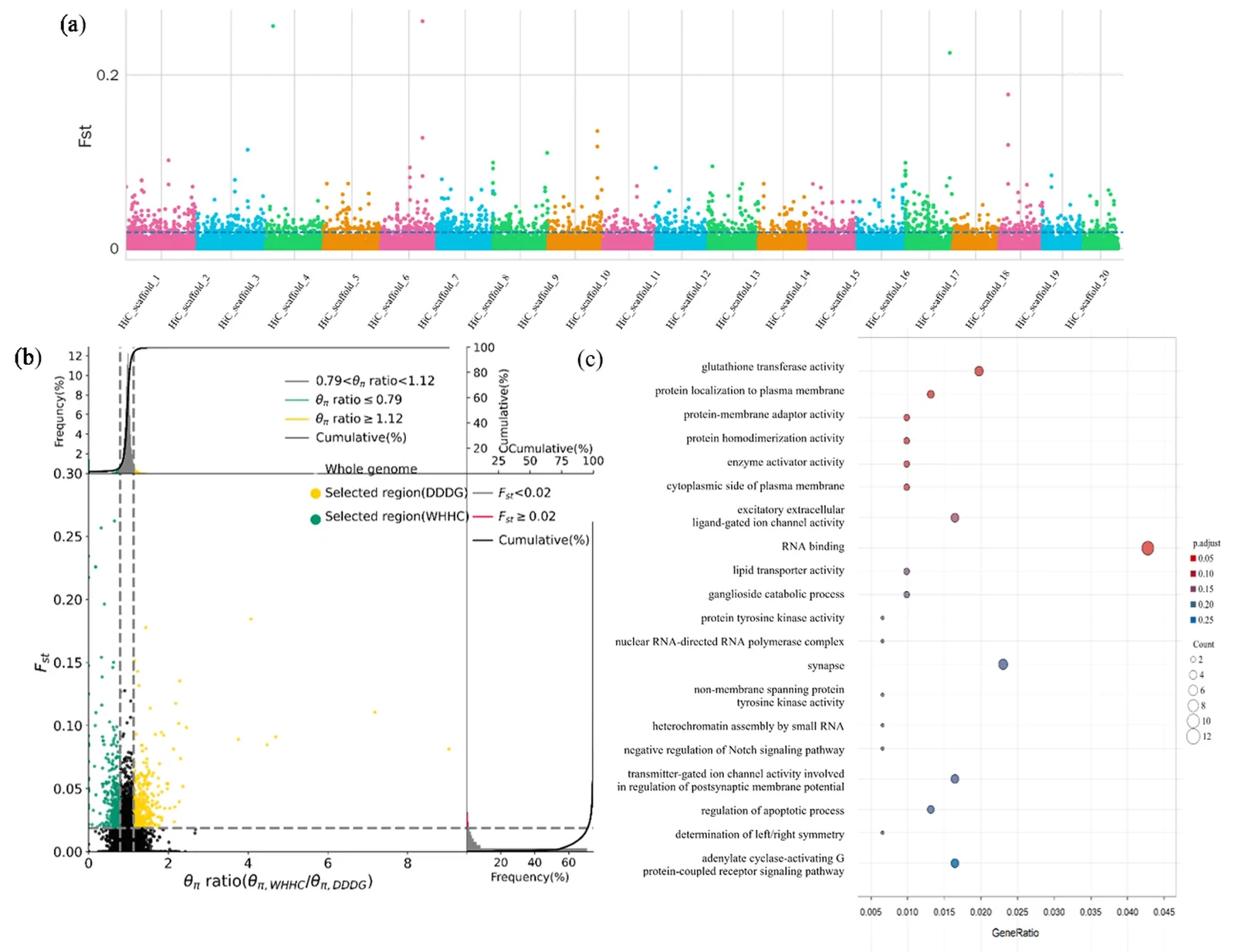
Selective sweep signals and GO enrichment analysis of *S. purpuratus* (WHHC vs. DDDG). (**a**) The values of Fst between WHHC and DDDG were plotted against the position on each HiC_scaffold. (**b**) Distribution of Fst values and θπ ratio calculated between WHHC and DDDG. (**c**) GO term analysis of selected genes in WHHC and DDDG.

We searched for signatures of localized selection along the temperature–salinity gradient using two genome-wide scans with DDDG as the focal population. The cross-sea comparison with YTCD identified 381 candidate regions and 775 genes (Table S4), concentrated on HiC_scaffold_1, 2, 8, 10, and 12 (Figure 4a,b). These genes were enriched in immune responses, transmembrane transport, and cell–extracellular matrix adhesion. After exclusion of the histone gene cluster (g13820-g13861), the top GO terms were dominated by membrane- and signaling-related functions (Figure 4c; Table S5). These included protein–membrane adaptor activity (GO:0043495), signaling receptor activity (GO:0038023), apical plasma membrane (GO:0016324), collagen-containing extracellular matrix (GO:0062023) and transmembrane transporter activity (GO:0022857), together with the Toll-like receptor signaling pathway (GO:0002224; seven genes). TLR-pathway genes showed elevated differentiation in DDDG under the cross-sea reference. The within-sea comparison with WHHC identified 365 candidate regions and 690 genes (Table S6), concentrated on HiC_scaffold_2, 5, 6, 12, and 16 (Figure 5a,b). Candidate genes from this comparison spanned membrane–surface and extracellular interactions, receptor- and ion-channel-mediated signal transduction, and detoxification and lipid metabolism. The most significantly enriched terms (Figure 5c, Table S7) included protein–membrane adaptor activity (GO:0043495), protein localization to the plasma membrane (GO:0072659), glutathione transferase activity (GO:0004364), lipid transporter activity (GO:0005319), synapse (GO:0045202), and excitatory extracellular ligand-gated ion channel activity (GO:0005231).

Across the two reference comparisons, a total of 1268 candidate genes potentially affected by selection were identified, of which 197 were detected in both (Figure S3a). These candidate regions were corroborated by the XP-CLR scan: they were significantly enriched among windows in the genome-wide top 5% of XP-CLR scores in both comparisons (13.4% of candidate windows versus 5% expected, a 2.7-fold enrichment, *p* = 3.4 × 10^−14^ for DDDG vs. WHHC; 10.5% versus 5%, a 2.1-fold enrichment, *p* = 5.1 × 10^−7^ for DDDG vs. YTCD) (Figure S4a–c). Among the 197 consensus genes (Figure S3b), 42 belonged to the tandem histone gene cluster (g13820-g13861) and were excluded before downstream analysis. Protein–membrane adaptor activity (GO:0043495) ranked first in the GO enrichment results of both comparisons and represented the strongest and most consistent functional signal across references. The remaining 155 non-histone candidate genes were assigned to five functional categories (Figure S3c). These were receptor and signal transduction, membrane transport and ion channels, innate immunity and defense, extracellular matrix and adhesion, and detoxification and metabolism. The candidate regions detected in both comparisons contained TLR6, TLR13, complement-related factors, and the antiviral response genes TRIM33 and guanylate binding protein 1. An NHERF1-like and a periostin-like extracellular matrix protein were also present. Functional annotations linked these genes to immune recognition, signal transduction, extracellular matrix organization, and shell biomineralization.

## 3. Discussion

Unlike earlier mitochondrial and transcriptomic studies, which were restricted to maternally inherited loci, marker variation, or gene expression, the present genome-wide SNP data resolve nuclear population structure and reveal candidate differentiated regions that were previously inaccessible.

### 3.1. Low Genetic Diversity and Its Potential Causes

The genetic diversity of *S. purpuratus* is comparable to that of other burrowing bivalves in the Yellow and Bohai Seas, such as *Scapharca broughtonii* [23] and *Solen grandis* [24]. It is lower than that reported for *Chlamys farreri* [3], *Mizuhopecten yessoensis* [18], and *Crassostrea gigas* [19]. This low diversity reflects both the ecological and life-history traits of *S. purpuratus* and the regional history. As a stenohaline, cold-water, burrowing species, it is confined to high-latitude, low-temperature muddy–sandy seafloor [10,11], so its historical range and effective population size have long been constrained by the availability of suitable habitat. Quaternary sea-level changes also reshaped the continental-shelf habitats of the Yellow and Bohai Seas [8,25]. Our PSMC results revealed a long-term decline in effective population size in all five populations after a Middle Pleistocene expansion. Historical habitat contraction is consistent with the currently limited genetic diversity. Without historical samples, the contributions of long-term demographic history and recent fishing pressure cannot be separated [26]. These cross-species comparisons are approximate, because the cited studies differ in SNP filtering, genome assembly quality, and sampling coverage. Moreover, all estimates depend on the reference assembly used for read mapping [27]. The values therefore indicate relative rather than absolute diversity.

All five populations showed positive Fis, and Ho was lower than He; a general heterozygote deficiency was thus apparent in every population. This could stem from inbreeding, but could also reflect the concentrated sampling of related individuals [7], unequal reproductive contributions among broodstock [9,28], or cryptic population substructure [14]. The lowest genetic diversity and the highest Fis coincided in YTLZ, whose elevated Fis could reflect a small effective number of breeders, related individuals, or technical artifacts such as uneven depth and reference bias. The present data cannot distinguish among these explanations, although multi-year sampling across Laizhou Bay together with kinship estimates would help resolve the question [29].

Quantitative traits and genetic diversity ranked the populations differently. Shell size and wet weight need not reflect population-level genomic variation. The trait differences among populations track water temperature, salinity, and substrate type [6,11,20].

### 3.2. Population Connectivity and Inferred Historical Allele Exchange

Regional currents could transport planktonic larvae among populations in the Yellow Sea and Bohai Sea [30]. Such dispersal may limit the accumulation of allele-frequency differences across the Bohai Strait. Similar LD decay profiles are consistent with broadly comparable recombination and demographic backgrounds but do not demonstrate current gene flow. At the geographic scale examined, differentiation in *S. purpuratus* was lower than that reported for *Chlamys farreri* [3] and *Scapharca broughtonii* [23]. Larval duration, hydrodynamic transport, and parentage-based dispersal all bear on present connectivity [5].

Interregional trade in wild-caught clams could introduce individuals from one population into another if they enter local breeding or farming stocks [31]. This pathway was not documented for the populations sampled here. TreeMix identified two migration edges compatible with historical allele exchange, although these edges represent modelled covariance rather than observed migration. These covariance patterns may reflect historical exchange, larval dispersal, or human-mediated movement.

The weak differentiation is therefore unlikely to reflect the sampling strategy. The five sites span two seas and several hundred kilometres, well within the resolving power of genome-wide SNPs, and, as described in Section 4.1, were chosen to span contrasting hydrographic conditions, so an under-sampled range can be excluded. It is likewise unlikely to be an artefact of the analytical approach: the pairwise Fst values (0.0035–0.0040) were small, but their bootstrap 95% confidence intervals excluded zero (Table S3), indicating weak rather than absent differentiation, and the NJ tree resolved few stable nodes, independently corroborating the absence of discrete groups. The low genome-wide diversity (Section 3.1) reduces the signal available to clustering and may partly account for the lack of a clear K > 1 structure; this is a limitation of the data rather than evidence against connectivity. Of the remaining biological alternatives—unequal reproductive contributions [9], cryptic substructure [14], and recent or human-mediated movement [15]—none can be fully excluded, but the observed pattern most plausibly reflects sustained gene flow maintained by larval dispersal along regional currents, with localized differentiation confined to DDDG. The five sampling locations are therefore best interpreted as demes within a single, highly connected population continuum rather than as reproductively isolated populations.

The five sites span a gradient from an estuarine, cold-water northern site (DDDG) to a semi-enclosed, seasonally more variable southern embayment (YTLZ). Environmental heterogeneity contrasts with the near-absence of genome-wide genetic structure. The environmental gradient alone has therefore not driven genome-wide divergence, even though localized candidate signals were detected in DDDG. Because temperature, salinity, and pathogen load were not measured at the sites, these signals cannot yet be attributed to any specific environmental variable.

### 3.3. Localized Candidate Differentiated Regions

Selection scans recovered localized differentiation signals in DDDG in both reference comparisons, coexisting with genome-wide homogenization. The same combination is documented in *Mytilus coruscus* [2], *Anadara granosa* [15], and *Crassostrea virginica* [32], where local adaptation persists under high gene flow [30,33,34].

The consensus genes included one tandem histone gene cluster (g13820-g13861). Such high-copy-number tandem repeats are prone to systematic biases in SNP detection and population-differentiation statistics, producing selection-like signals [35,36]; this cluster was therefore excluded from downstream analyses. The number and chromosomal location of such clusters vary among venerid clams [37], further arguing against their treatment as stable biological signals.

After removal of the tandem histone gene cluster, the 155 non-histone consensus genes were annotated and subjected to GO enrichment. These consensus genes were enriched in receptor and signal transduction, membrane transport and ion channels, innate immunity and defense, extracellular matrix and adhesion, and detoxification and metabolism (Section 2.6). TLR6, TLR13, complement-related factors, and antiviral effectors participate in pathogen recognition and immune defense [8,38]. Transmembrane transporters, ion channels, and membrane–extracellular adaptor proteins contribute to ion homeostasis and cellular signal transduction [8]. Extracellular matrix proteins take part in tissue adhesion and shell formation [22,39,40]. These functional categories implicate low temperature, salinity change, pathogen exposure, and ion-homeostasis regulation as candidate environmental drivers. For the reasons given in Section 4.1, DDDG was treated as the focal population. This study did not measure temperature, salinity, or pathogen load at the sampling sites, nor did it validate candidate gene function, so these functional categories are best regarded as hypotheses for future genotype–environment association studies, which will require synchronous environmental monitoring and a broader sampling range. They are not evidence of adaptive divergence.

The Fst and nucleotide-diversity-ratio scan detected anomalous genomic regions, but this alone does not constitute direct evidence of adaptive function. Similar signals can also arise from recombination-rate variation, gene density, structural variation, or demographic history [32,35]. The fitness effects of these regions remain to be tested by genotype–environment association and functional expression assays over a broader sampling range [32].

### 3.4. Implications for Germplasm Conservation and Sustainable Use

For resource conservation, the low differentiation among the five populations supports their management as a single germplasm unit [14,16]. Candidate differentiated regions in DDDG and the elevated Fis in YTLZ warrant separate monitoring [1,23].

Stock enhancement should use diverse, weakly related broodstock. Increasing the effective number of breeders and balancing sex ratios and family contributions would limit inbreeding and genetic drift [29,41]. Genomic markers can trace the genetic contribution of released individuals to wild populations, refining release strategies [7,9,21].

For selective breeding, low differentiation permits the assembly of a base population from multiple geographic sources [42]. Outbreeding depression and loss of local adaptation should be assessed before assembly [30]. The low genetic diversity would limit long-term selection response; base-population construction should prioritize broadening the genetic basis over retaining a few phenotypically superior individuals [4,17,25]. Candidate genes in DDDG related to immune defense, membrane transport, detoxification and metabolism, and shell formation constitute targets for the functional evaluation of stress tolerance and growth-related traits.

## 4. Materials and Methods

### 4.1. Sample Collection

**Figure 6:**
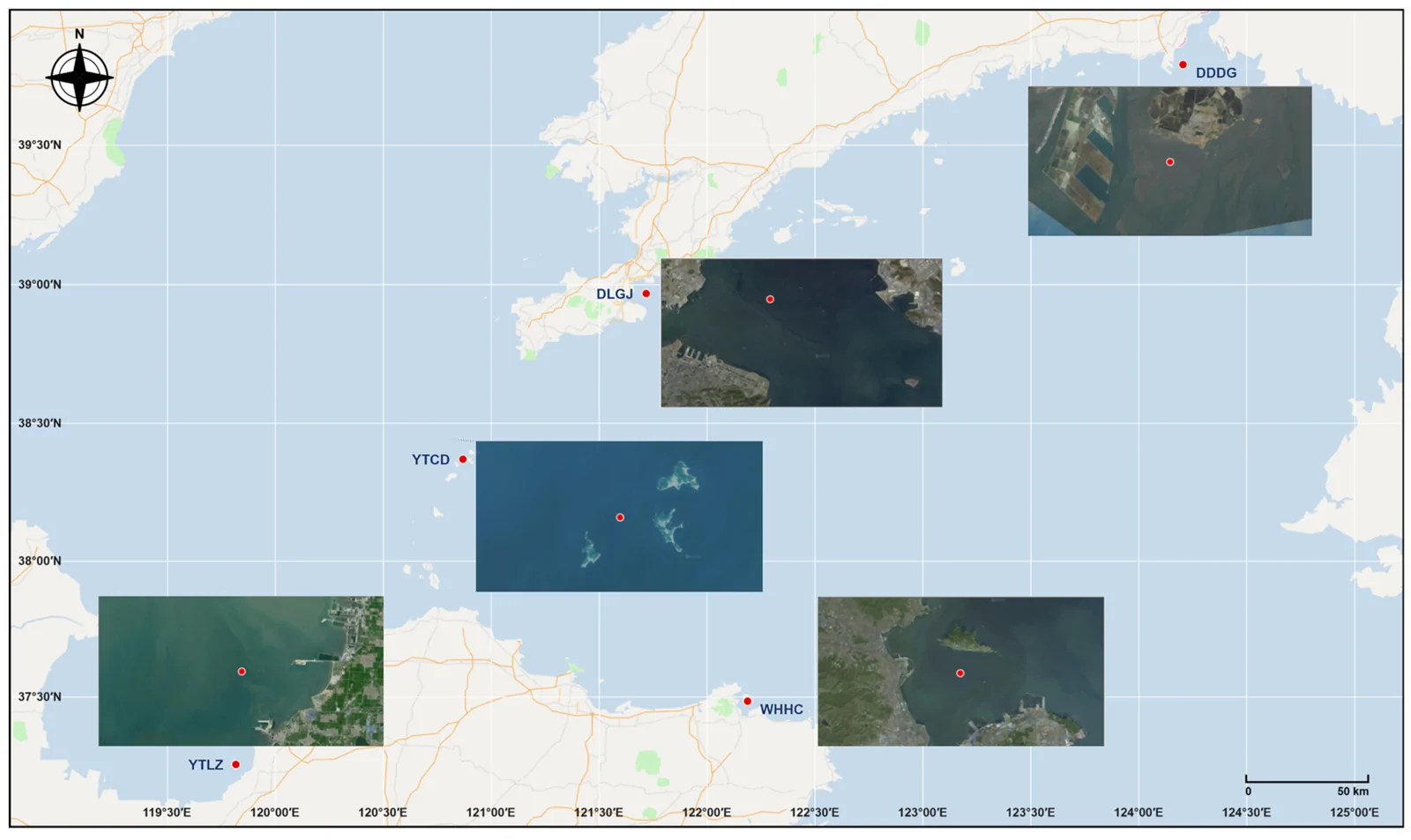
Sampling locations and local environment of the five *S. purpuratus* populations.

Sampling was conducted at DDDG, DLGJ, WHHC, YTCD, and YTLZ between November and December 2025, with 15 wild *S. purpuratus* collected per population (Figure 6; Table S8). These five populations were selected to represent contrasting hydrographic conditions. They span approximately 2.5° of latitude (37°15′ N–39°47′ N) and 4.4° of longitude (119°49′ E–124°12′ E). DDDG lies at the northernmost limit of the sampling range, in the northern Yellow Sea near the Yalu River estuary. There, freshwater discharge lowers salinity and winter sea-surface temperatures are the lowest among the sites. DLGJ and WHHC are open-coast northern Yellow Sea sites with cooler, higher-salinity water. YTCD is an island site at the mouth of the Bohai Sea (Bohai Strait), a major corridor for larval exchange between the Yellow and Bohai Seas. YTLZ lies within Laizhou Bay, a semi-enclosed, shallow embayment of the Bohai Sea with reduced tidal flushing and a comparatively large seasonal temperature and salinity amplitude.

Quantitative traits were measured with a vernier caliper (Mitutoyo, Kawasaki, Japan) before the adductor muscle was excised and frozen in liquid nitrogen for DNA extraction. All 75 individuals were wild, sexually mature adults aged 3 years and of market size. No juvenile or larval specimens were included, which avoided confounding by age- and stage-related genetic variation.

### 4.2. Resequencing

Following the protocols of Lv et al. [18], genomic DNA was extracted and libraries were constructed. Libraries with a mean insert size of ~350 bp were sequenced as 150 bp paired-end (PE150) reads on the BGI DNBSEQ platform (BGI-Shenzhen, China) to a target average depth of ~10×.

### 4.3. Read Mapping and Variant Calling

Raw reads were quality-filtered with fastp v0.23.4 [43]. Adapter sequences were removed, and low-quality bases were trimmed from the 5′ and 3′ ends by sliding-window trimming. Reads were discarded based on four criteria: more than five unidentified bases (N), more than 40% of bases with a quality below Q15, a mean base quality below Q20, or a trimmed length below 50 bp. Clean reads were aligned to the Hi-C scaffold-level reference genome of *S. purpuratus* [27] (2175 scaffolds; N50 = 52.4 Mb; https://doi.org/10.6084/m9.figshare.20400975.v1) with BWA-MEM2 v2.2.1 using the ‘mem –R’ option and otherwise default parameters. The resulting BAM files were sorted, deduplicated, and indexed with SAMtools v1.19 and Picard v2.27.5. Variants were called with GATK v4.6.0.0 following the per-sample gVCF pipeline (HaplotypeCaller, CombineGVCFs, GenotypeGVCFs) [44]. Raw variants were hard-filtered with GATK VariantFiltration. SNPs were filtered with FS > 60, ReadPosRankSum < −8.0, QUAL < 30, MQ < 40, MQRankSum < −12.5 and SOR > 3; indels were filtered with FS > 200, ReadPosRankSum < −20.0 and QUAL < 30. Biallelic SNPs with a minor allele frequency (MAF) > 0.05 and a genotyping rate ≥ 80% (missingness < 20%) were then retained with VCFtools v0.1.17 for downstream analysis, using the settings –maf 0.05 –max-missing 0.8–min-alleles 2 –max-alleles 2 –remove-indels [45]. The retained SNPs were functionally annotated with ANNOVAR v20200607.

### 4.4. Population Genetic Diversity and Linkage Disequilibrium

As described by Yao et al. [16], observed heterozygosity (Ho), expected heterozygosity (He), polymorphic information content (PIC), inbreeding coefficient (Fis), and minor allele frequency (MAF) were calculated for each population with VCFtools. Linkage disequilibrium (LD) was estimated as the squared correlation coefficient (r^2^) between pairs of biallelic SNPs using PopLDdecay v3.42 with default parameters (maximum distance 300 kb). The decay distance was defined as the physical distance at which r^2^ decays to half of its maximum value.

### 4.5. Population Genetic Structure and Differentiation

A neighbor-joining (NJ) tree was built from pairwise genetic distances among individuals in PHYLIP v3.696 [46] with 1000 bootstrap replicates, and principal component analysis (PCA) was performed in GCTA v1.93.2. Population structure was inferred with ADMIXTURE v1.3.0 for K = 1–10, with the optimal K selected according to the lowest cross-validation error [2,15]. Pairwise fixation index (Fst) among populations was computed in VCFtools. The statistical significance of each pairwise Fst value was assessed with a window-based bootstrap (1000 replicates over 50-kb genomic windows). An Fst value was considered significant when its 95% confidence interval excluded zero. To reduce the effect of LD on structure inference, SNPs were pruned using a 50 kb window and a threshold of r^2^ > 0.2 before PCA and ADMIXTURE.

### 4.6. Gene Flow and Historical Effective Population Size

Gene flow among populations was inferred with TreeMix v1.3.1, testing 1–6 migration events (m) and selecting the optimal m by the Δm statistic in OptM v0.1.6. Model fit was evaluated from the residual matrix as in Mao et al. [19]. Historical effective population size (Ne) was estimated with PSMC v0.6.5-r67 [47] from one or two individuals per population, following He et al. [7]. A generation time of 3 years and a mutation rate of 4.5 × 10^−8^ per site per generation were assumed.

### 4.7. Genome-Wide Detection of Selection Signals

DDDG was chosen as the target because it occupies the most environmentally distinct of the five sites. It is therefore, a priori, the population most likely to harbor localized candidate differentiation signals. WHHC, which shares the Yellow Sea with DDDG, served as a within-sea reference for evaluating differentiation between adjacent populations. YTCD, located in the Bohai Sea, served as a cross-sea (Yellow Sea-Bohai Sea) reference. Genome-wide selective sweep scans and candidate-region delineation followed Wang et al. [3]. Pairwise weighted Fst and the nucleotide diversity ratio (π ratio) were computed in sliding windows of 50 kb with a 25 kb step, a window size that balances genomic resolution against single-locus noise in Fst and π. Candidate regions were defined as the intersection of windows in the top 5% of both the weighted Fst and the π-ratio distributions. Requiring agreement between two complementary statistics reduces the false-positive rate relative to any single-statistic scan. Genome-wide XP-CLR (cross-population composite likelihood ratio) scores were additionally computed for the same two comparisons as a complementary scan [48]. The significance of the overlap between the candidate windows and the genome-wide XP-CLR top 5% was assessed with a hypergeometric test. The tandem histone gene cluster was identified by NR annotation as a contiguous block of histone genes (H1, H2A, H2B, H3 and H4) on a single scaffold (gene IDs g13820-g13861, *n* = 42). Such high-copy-number tandem repeats are prone to systematic errors in read mapping, SNP calling and differentiation statistics, so this cluster was masked prior to functional annotation.

### 4.8. Functional Annotation and Enrichment Analysis

Candidate-region genes were functionally annotated by homology against the NCBI non-redundant protein (NR) database and assigned Gene Ontology (GO) terms, using the annotation of the same reference genome [27]. GO enrichment was performed using all GO-annotated genes of that annotation as the background (*n* = 18,262), with the Benjamini–Hochberg correction (q < 0.05). Both analyses were performed on the refined non-histone candidate set (*n* = 155).

## 5. Conclusions

Whole-genome resequencing of 75 wild *S. purpuratus* individuals from five populations across the Yellow and Bohai Seas uncovered relatively low genetic diversity, heterozygosity deficiency, and weak population differentiation. No genetic structure consistent with geography was detected under the present sampling and marker resolution; the small but statistically significant differentiation is consistent with a highly connected population continuum sustained by larval dispersal along regional currents. DDDG carried localized candidate differentiation signals that recurred in both reference comparisons, and the underlying genes were enriched in immune defense, membrane transport, and detoxification pathways. The five populations are best managed as a single regional unit, with DDDG and YTLZ warranting separate monitoring. Selective breeding should prioritize a broad genetic base to sustain long-term selection response.

## Figures and Tables

**Table 1:** Summary statistics of quantitative traits for five *S. purpuratus* populations.

Population	Shell Height (mm)	Shell Length (mm)	Shell Width (mm)	Shell Height/Shell Length	Shell Width/Shell Length	Wet Weight (g)
DDDG	72.54 ± 3.10	92.18 ± 3.70	47.17 ± 2.75	0.787	0.512	205.34 ± 19.52
DLGJ	76.63 ± 4.50	93.80 ± 5.82	51.52 ± 2.28	0.817	0.549	252.30 ± 31.12
YTCD	71.11 ± 3.13	90.65 ± 4.63	47.84 ± 2.50	0.784	0.528	204.52 ± 28.99
WHHC	80.32 ± 3.43	102.27 ± 3.29	53.74 ± 3.06	0.785	0.525	271.59 ± 37.58
YTLZ	79.59 ± 2.95	101.71 ± 3.85	54.64 ± 2.06	0.783	0.537	251.26 ± 28.65

**Table 2:** Statistics of genetic parameters for five *S. purpuratus* populations.

Population	MAF	Ho	He	PIC	Fis
DDDG	0.0713	0.1257	0.1324	0.1236	0.0506
DLGJ	0.0708	0.1249	0.1312	0.1229	0.0480
YTCD	0.0701	0.1213	0.1304	0.1219	0.0698
WHHC	0.0704	0.1241	0.1309	0.1223	0.0519
YTLZ	0.0699	0.1193	0.1301	0.1215	0.0831

## Data Availability

The raw whole-genome resequencing data generated in this study have been deposited in the Genome Sequence Archive (GSA) of the National Genomics Data Center (NGDC), China National Center for Bioinformation (CNCB), under BioProject accession CRA051397 (https://ngdc.cncb.ac.cn/gsa/browse/CRA051397) (accessed on 11 October 2026). All other data supporting the findings are included in the manuscript and Supplementary Materials.

## Supplementary Materials

The following supporting information can be downloaded at: https://www.mdpi.com/article/10.3390/ijms27219627/s1.
