# Supplementary material for: Whole Genome Resequencing Reveals High Genetic Homogeneity and Localized Candidate Differentiation Signals in Saxidomus purpuratus Along the Yellow and Bohai Sea
Source: Int J Mol Sci. 2026 Oct 27;27(21):9627. doi: 10.3390/ijms27219627 (PMC13762793; doi:10.3390/ijms27219627)

Table S1. Summary of sequencing data.

Population	Raw bases (bp)	Clean bases (bp)	GC content (%)	Mapping rate (%)	Average depth (X)
DDDG	174085172400	172714836090	35.25	98.03	10.77
DLGJ	168951234600	167739987345	34.98	97.97	10.44
WHHC	152303922600	151534569735	34.92	97.63	9.43
YTCD	151471417800	150717926295	34.93	97.67	9.39
YTLZ	163234568700	161994421455	35.60	96.32	9.89

Table S2. Genomic distribution and functional annotation of the SNPs retained for the population-genetic analyses (n = 20,702,921).

Category	Number of SNPs
Intergenic	11,073,890
Intronic	6,789,870
Exonic	1,216,750
Downstream	869,638
Upstream	729,905
Upstream; downstream	21,941
Splicing	927
**Total**	**20,702,921**
Exonic Synonymous	655,798
Exonic Non-synonymous	552,121
Exonic Stopgain	7,651
Exonic Stoploss	1,179
Exonic Unknown	1

Table S3. Population differentiation index (Fst) between two of the five populations.

	DDDG	DLGJ	WHHC	YTCD	YTLZ
DDDG	-	*	*	*	*
DLGJ	0.00350	-	*	*	*
WHHC	0.00377	0.00372	-	*	*
YTCD	0.00370	0.00370	0.00367	-	*
YTLZ	0.00378	0.00385	0.00367	0.00398	-

Statistical significance was assessed with a window-based bootstrap (1,000 replicates of 50-kb genomic windows); the 95% confidence interval of every pairwise Fst excluded zero. *$P < 0.05$.

Table S8. Geographic coordinates of different populations.

Population	Longitude	Latitude
YTLZ	119°49′	37°15′
YTCD	120°52′	38°22′
DLGJ	121°43′	38°58′
DDDG	124°12′	39°47′
WHHC	122°11′	37°29′

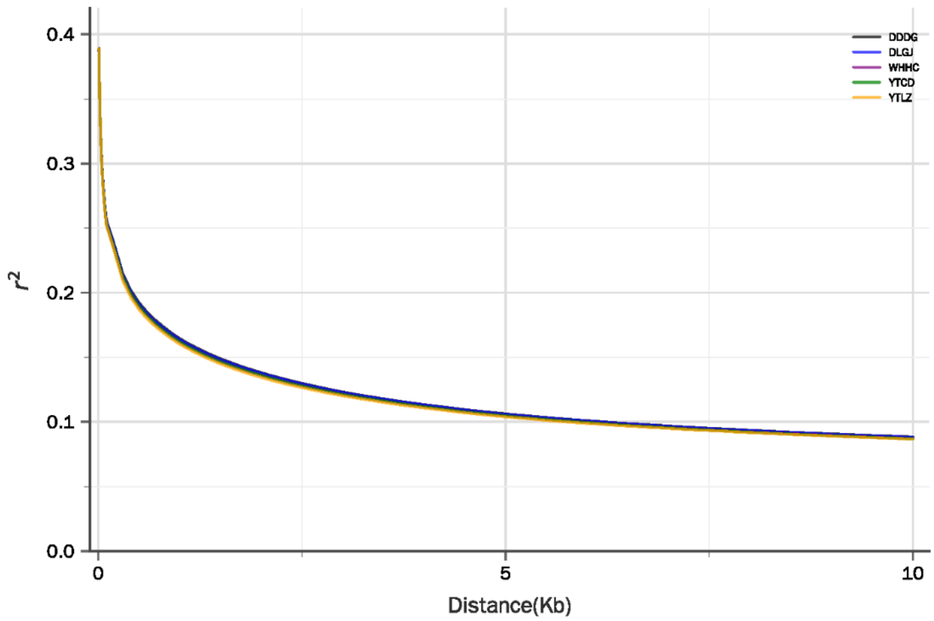

Figure S1. LD decay curve of five *S. purpuratus* populations.

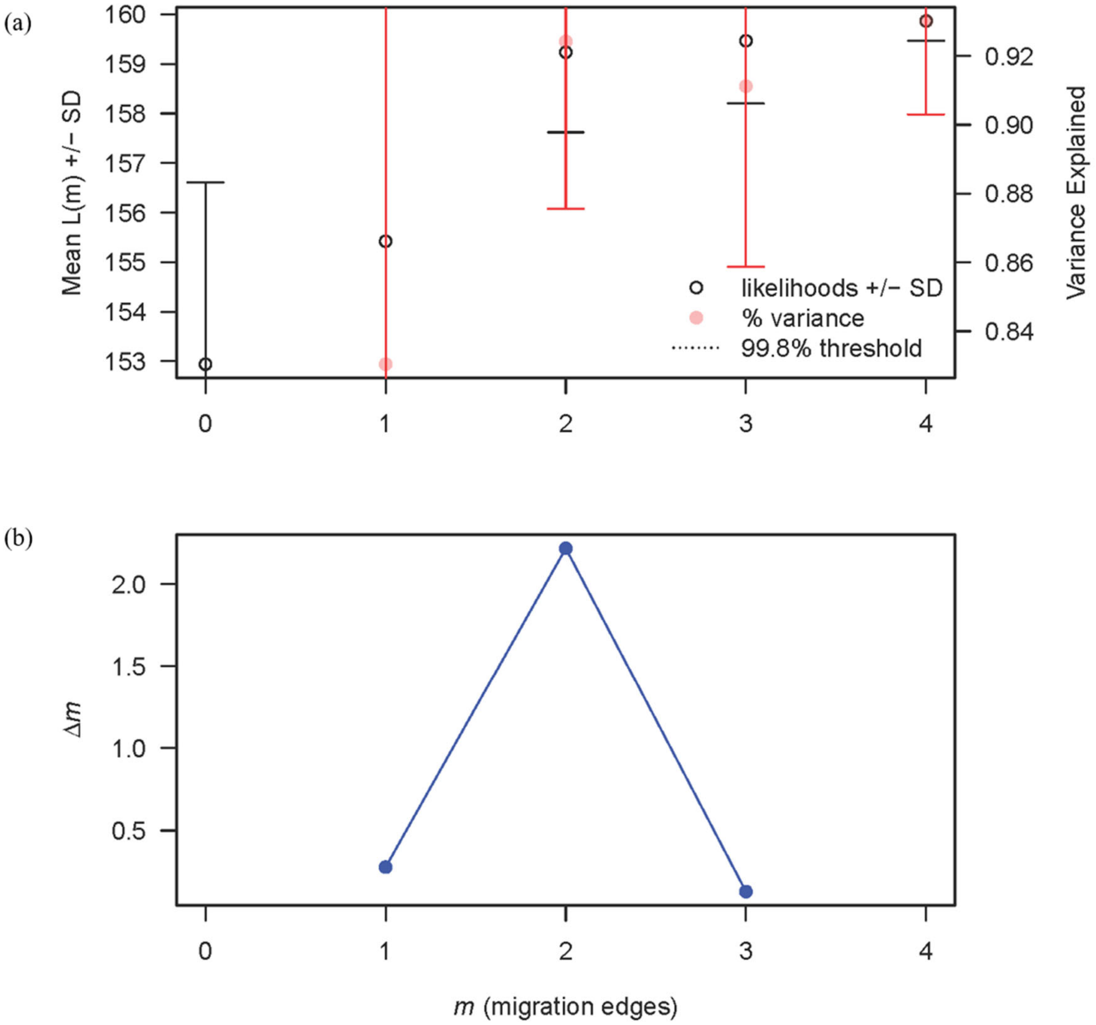

Figure S2. Evaluation of the optimal m value. (**a**) Analysis of covariance. (**b**) Determination of the optimal m value.

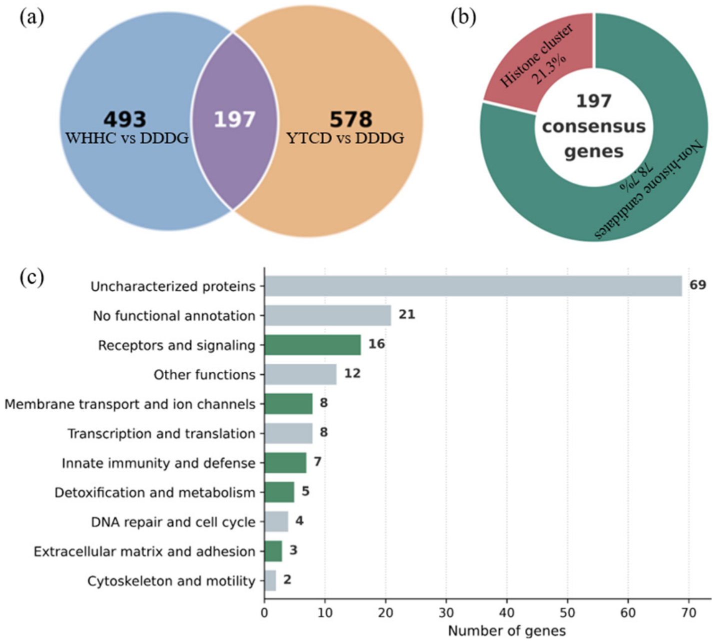

Figure S3. Consensus candidate genes identified under two independent reference populations of *S. purpuratus*. (**a**) Venn diagram showing the overlap of candidate genes detected with WHHC and YTCD as reference populations. (**b**) Composition of the 197 consensus genes, comprising a tandem-repeat histone cluster (g138xx, n = 42, 21.3%) that represents a technical artifact and 155 non-histone candidates (78.7%) retained for biological interpretation. (**c**) Functional classification of the 155 non-histone candidates based on NR annotations.

Figure S4. XP-CLR scans corroborating the Fst/π candidate regions in *S. purpuratus*. Grey points, all windows; red points, the candidate windows defined by the top 5% of both Fst and the π-ratio. (**a**) Genome-wide XP-CLR distribution for DDDG vs WHHC, computed in 50-kb windows (537 for this comparison); dashed line, genome-wide top 5% XP-CLR threshold (2.79); (**b**) Genome-wide XP-CLR distribution for DDDG vs YTCD (497 candidate windows; threshold 2.91); (**c**) Percentage of the candidate windows falling within the genome-wide XP-CLR top 5% (error bars, 95% CI); dashed line, 5% expected by chance. Enrichment was significant in both comparisons (2.7-fold, $P = 3.4 \times 10^{-14}$ and 2.1-fold, $P = 5.1 \times 10^{-7}$; hypergeometric test).

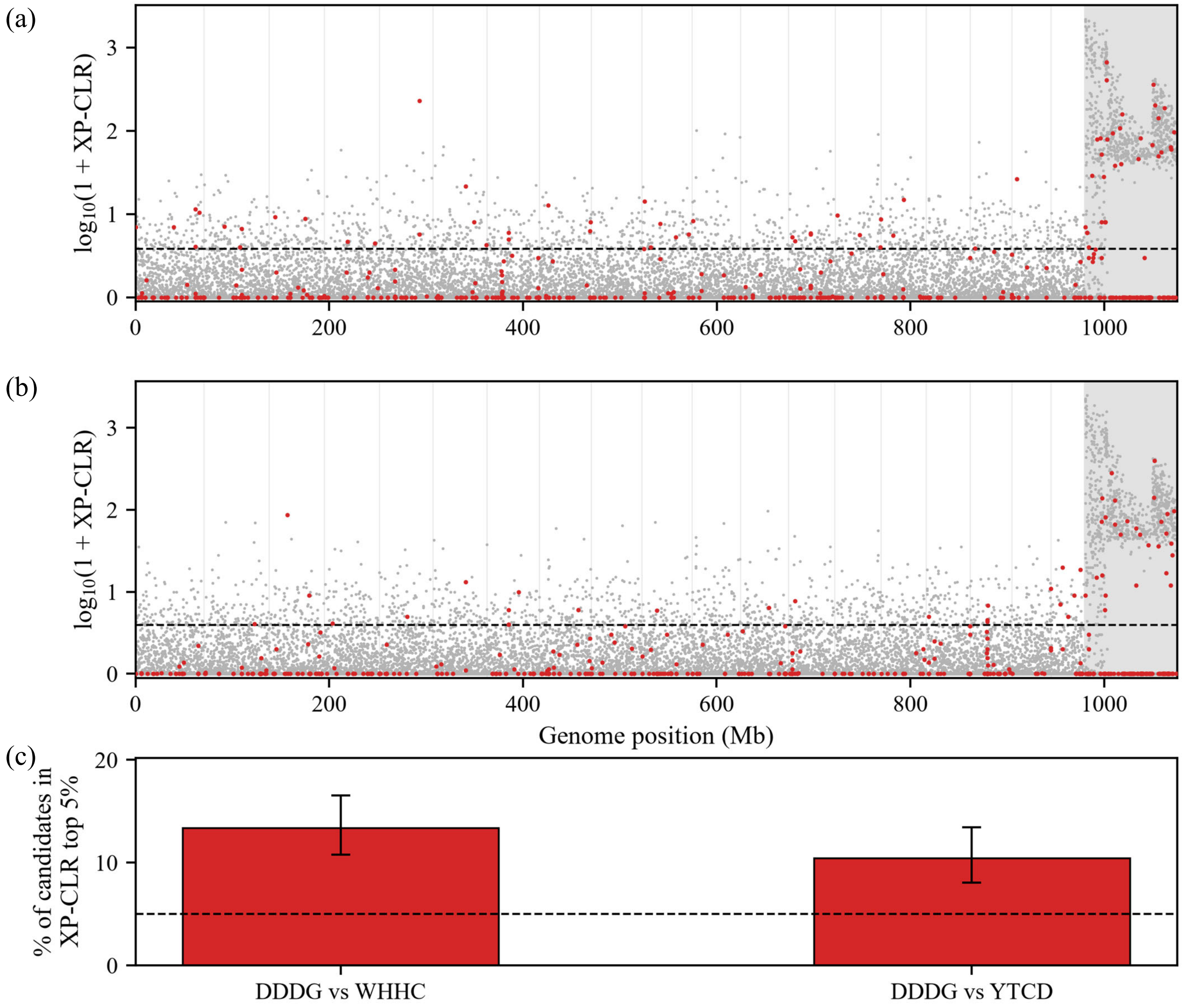

Supplement: Supplementary file 1 [file ijms-27-09627-s001.zip › Supplementary Figures and Tables.pdf]
